# Is canaloplasty with mitomycin c a safe procedure in myopic glaucoma?

**DOI:** 10.1007/s00417-022-05655-0

**Published:** 2022-04-18

**Authors:** Vivienne Dooling, Alexandra Lappas, Thomas Stefan Dietlein

**Affiliations:** Department of Ophthalmology, Faculty of Medicine and University Hospital Cologne, University of Cologne, Kerpener Str. 62, 50937 Cologne, Germany

**Keywords:** Canaloplasty with Mitomycin C, Myopia, Trabeculectomy, Filtrating glaucoma surgery, Success rates

## Abstract

**Purpose:**

Myopic glaucoma patients display a considerable risk of complications following antiglaucomatous filtering surgery, e.g., trabeculectomy. Canaloplasty with mitomycin C may reduce this risk by avoiding massive overfiltration.

**Methods:**

We performed retrospective analysis of 31 eyes with myopia that underwent canaloplasty modified with mitomycin C in a consecutive single-surgeon case series. Annual data and success rates were analysed. Twenty-three myopic eyes that had received conventional trabeculectomy with mitomycin C were recorded as a comparison.

**Results:**

The 31 eyes with a follow-up of 40 ± 26 months after canaloplasty had a mean spherical equivalent of − 8.4 ± 4.5 dioptres. Intraocular pressure decreased from 32.3 ± 9.6 mmHg (range: 17 to 58) to 16.8 ± 8.1 mmHg (range: 5 to 44) 1 year after surgery (− 46%; *p* < 0.001) with a medication score reduction from 5 to 1.2 (*p* < 0.001). Qualified success rates (Criterion B: no revision surgery, IOP < 21 mmHg, IOP reduction > 20%) were 83% after 1 year and 61% at the 2nd and 3rd years. In 5 eyes (16%), early ocular hypotony (≤ 5 mmHg) was observed. Two eyes (7%) showed transient choroidal detachment and swelling. The 23 eyes that had received trabeculectomy had success rates (Criterion B) of 91% at the 1st and 86% at the 2nd and 3rd years. Hypotony occurred in 10 eyes (44%), and 4 eyes (17%) showed choroidal detachment or macular folds.

**Conclusions:**

Postoperative complications related to overfiltration were less frequent after canaloplasty with mitomycin C. Midterm data proved good efficacy. Pressure reduction, success rates and rates of medication free patients were significantly higher in trabeculectomy compared to modified canaloplasty with mitomycin C.


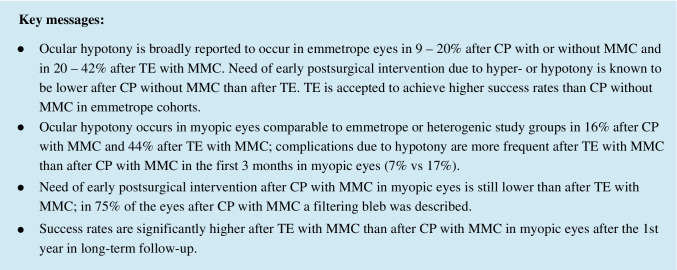


## Introduction

Myopic glaucoma patients are considered to be at higher risk for ocular hypotony following traditional filtrating glaucoma surgery, resulting in choroidal detachment and hypotony maculopathy [1–4]. Canaloplasty (CP) has a lower risk for overfiltration than trabeculectomy (TE) by not perforating the anterior chamber. But it can create a subconjunctival filtering zone, which can be sustained by application of mitomycin C (MMC). The antifibrotic agent’s contribution to intraocular pressure (IOP) reduction as well as its safety is broadly reported on [5]. As a consequence, CP with MMC as non-penetrating procedure may be considered as an alternative to TE with MMC in glaucoma patients with myopia. TE with MMC reaches IOP reductions of 43–59% after 12 months with mean IOP reported mostly between 10 and 14 mmHg [6–9], while CP ranges between a reduction of 22 and 56% with 13 to 16 mmHg after 12 months [6–12]. In this analysis, we compared CP with MMC to TE with MMC in a cohort of myopic patients.

## Methods

In this retrospective single-surgeon study, we analysed a case series of myopic patients <  − 2 dioptres (dpt) that needed surgical glaucoma treatment and that consecutively received CP with MMC at the University Hospital Cologne. Eyes which received CP without MMC or intraoperative conversion from canaloplasty to trabeculotomy were excluded. A second group of myopic patients that had received TE with MMC by the same surgeon predominantly in the time period before introduction of CP was analysed as a historical comparison. Study design and data collection was in accordance to the local ethical committee guidelines and the tenets of Helsinki.

### Examinations

Demographic and examination data were collected at baseline and postoperatively in intervals of 1 week, 3 months and further on annual follow-ups. Due to the retrospective design, there were ranges in the annual follow-up intervals with a maximum of 6 months. Visual acuity was converted from Snellen to logMAR. Medication scores were accounted after a modified table from Jacobi and Krieglstein [13, 14].

### Surgical procedure

The surgical procedures were performed by one experienced surgeon (TSD) either in general anaesthesia or in subconjunctival anaesthesia using lidocaine 2%. A fornix-based conjunctival flap was dissected. MMC was used in a concentration of 0.2 mg/ml and applied via two sponges for 2–3 min. In CP, the incision of the conjunctiva was followed by the preparation of a scleral flap just like in TE. Contrary to TE, no perforation of the anterior chamber took place, but by dissecting a second deep scleral flap, Schlemm’s canal was opened. A microcatheter with an attached prolene suture was inserted into Schlemm’s canal and pushed forward all 360°; the prolene-9 sutures were then redrawn through SC lumen. While the catheter was withdrawn, viscoelasticum was injected to widen the canal. The suture ends were tied together to cause tension of the Schlemm canal’s inner walls and thereby widen it. The surgeon adjusted the flow of aqueous under the scleral flap by altering the tension on the 9–0 nylon single-button flap sutures. The filtering zone was sewed up with conjunctival 8–0 vicryl sutures.

### Data analysis

After collection of the data until last follow-up in the patient’s records, all eyes were categorized into qualified success (with and without antiglaucomatous medication) or failure according to four criteria A, B, C, D. Criteria were adapted from the Tube versus Trabeculectomy Study and the guidelines of the World Glaucoma Association [15, 16]. Criterion A was met as a success when no glaucoma revision surgery was indicated. Combined cataract glaucoma surgery was not classified as failure if they had preoperative IOP ≤ 13 mmHg. Eyes with failure according to criterion A were excluded from following annual analyses. Criterion B was met as success with no revision surgery and IOP < 21 mmHg and criterion C with no revision surgery and IOP < 18 mmHg; both criteria include > 20% IOP reduction compared to baseline IOP. Criterion D was met as success with no revision surgery, IOP ≤ 15 mmHg and IOP reduction of ≥ 40%. Baseline IOP was defined as IOP within 3 months prior to surgery that caused the indication for surgical treatment. Annual follow-ups as well as meantime follow-up records were examined. Data was analysed with IBM SPSS Statistics Version 26 and according to distribution tested with Student’s *t*-test, Wilcoxon matched pair and Mann–Whitney *U*-test. Categorial variables were tested with chi-quadrat or reported descriptively. Kaplan–Meier survival curves were designed and tested with log rank test. *p*-values lower than 0.05 were accepted as statistically significant. Data were analysed split in the two investigated surgical procedures and additionally split into subgroups of medium (< − 2 to >  − 6 dpt) and high myopia (≤ − 6 dpt).

## Results

### Patients

In the CP group, 31 eyes of 26 patients with a mean spherical equivalent (SE) of − 8.4 were included. In the TE group, 23 eyes of 16 patients with SE of − 7.5 were included. Demographic and baseline characteristics are presented in Table 1. Except mean follow-up time, pachymetry and duration of intraoperative MMC application, no characteristics were found to be significantly different. Therefore, we concluded a comparability of the groups.

**Table 1:** Demographic and baseline characteristics

	Canaloplasty with MMC	Trabeculectomy with MMC	
	*n* = 31 eyes (26 patients) mean ± SD (range)	*n* = 23 eyes (16 patients) mean ± SD (range)	*p*-value
Age	59.6 ± 12.5 (42 – 86)	54.4 ± 11.4 (36 – 71)	0.124
Sex (m/f)	10/16	11/5	0.057
Right eye/left eye	14/17	12/11	0.784
Spherical equivalent (dpt)	− 8.4 ± 4.5 (− 2,25 to − 21)	− 7.5 ± 3.7 (− 3—− 18)	0.456
- Medium myopia > − 6 (*n* = 7/9)	− 4.5 ± 1.4	− 4.4 ± 1	0.896
- High myopia ≤ − 6 (*n* = 24/14)	− 9.6 ± 4.4	− 9.6 ± 3.4	0.989
Pachymetry (µm)	539 ± 39	507 ± 35	0.004
Lens status (phakic/pseudophakic)	20/11	18/5	0.370
POAG–PEX–others (%)	71%–23%–6%	65%–17%–18%	0.696
Glaucoma history (years)	12 ± 10.5 (1–40)	11 ± 9.3 (0–37)	0.730
Prior glaucoma surgery (%)	13%	13%	0.291
Prior SLT/ALT/IO (%)	26%	22%	0.679
Perimetry MD (dB)	− 11.2 ± 7.3	− 7.9 ± 5.7	0.089
PSD (dB)	5.9 ± 2.6	5.6 ± 2.4	0.761
Visual acuity (logMAR)	0.31 ± 0.43	0.27 ± 0.56	0.761
IOP (mmHg)	32.3 ± 9.6 (17 – 58)	30.0 ± 8.3 (16–49)	0.368
Medication score	5 ± 2.2	4.9 ± 1.7	0.861
Follow-up (months)	40 ± 26 (1–97)	61 ± 34 (1–118)	0.010
Duration of MMC (2 / 3 min)	2.3 ± 0.5	3 ± 0	< 0.001

*SD* standard deviation, *m* male, *f* female, *POAG* primary open angle glaucoma, *PEX* pseudoexfoliative glaucoma, *SLT* selective laser trabeculoplasty, *ALT* Argon laser trabeculoplasty, *IO* iridectomy, *MD* mean deviation, *PSD* pattern standard deviation

### IOP and medication score

**Fig. 1:**
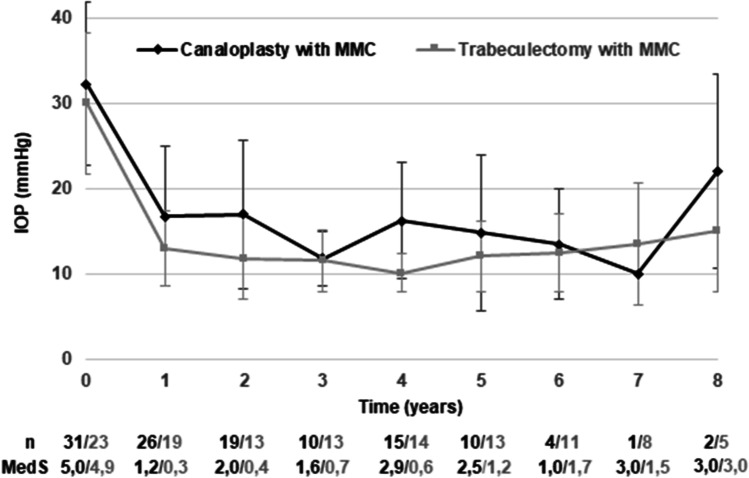
Mean IOP (± SD) after CP with MMC and TE with MMC at baseline and annual follow-ups, with *n* (number of eyes) and mean medication score (MedS). Black: canaloplasty with MMC, grey: trabeculectomy with MMC

After CP with MMC and TE with MMC, IOP and medication scores were significantly reduced to baseline in all annual follow-ups until the 5th (CP) and 6th (TE) years (Fig. 1). Significances of IOP reduction between the surgical procedures varied at the annual follow-ups. Reduction was significantly higher after TE than CP at 1st week (*p* = 0.04), 3rd month (*p* = 0.005), 2nd (*p* = 0.027) and 4th year (*p* = 0.001). No significant deviation was found at 1st (*p* = 0.086), 3rd (*p* = 0.738) and 5th year (*p* = 0.483). Homogeneity of the group was stronger in the TE group at every point.

After CP, the mean IOP of the highly myopic eyes was higher than that of the medium myopic group within the 1st year, but then constantly lower in 2nd, 3rd and 4th year. Further analysis of the subgroups showed that absolute and percental reduction in highly myopic eyes compared to their higher baseline IOP (33.4 ± 10.5 mmHg (*n* = 24)) were greater than that of the medium myopic eyes (baseline IOP: 28.3 ± 3.5 mmHg (*n* = 7)) throughout all follow-ups until 5th year. But there was no significance in this tendency (3rd month: *p* = 0.764, 1st year *p* = 1.000, 2nd year *p* = 0.262, 3rd year *p* = 0.067, 4th year *p* = 0.371, 5th year *p* = 0.711) with small subgroup sizes (highly myopic vs medium myopic: *n* at 1st year: 21 vs 5, 2nd year: 15 vs 4, 3rd year: 7 vs 3, 4th year: 10 vs 5, 5th year: 8 vs 2). After TE, analysis showed an alternating pattern in IOP reduction rates between the subgroups of medium and high myopia. The highly myopic group has lower mean IOPs in long follow-up though reduction compared to baseline is tendentially greater in medium myopic eyes. With similar baseline IOP and small subgroups, there is no significancy of difference at any follow-up (baseline IOP: medium myopia group, *n* = 9, 29.6 ± 7.2 mmHg; high myopia group, *n* = 14, 30.3 ± 9.1 mmHg).

In the CP group, 58% of the eyes were without medication after 1 year and 53%, 50%, 33% and 50% after 2 to 5 years, respectively. After TE, 90% of the eyes were without any medication after 1 year and 85%, 69%, 79% and 62% after 2 to 5 years, respectively.

### Visual acuity

After CP, baseline best corrected visual acuity decreased significantly at the 1st postoperative week to 0.58 ± 0.60 logMAR (*p* < 0.001), but recovered to 0.36 ± 0.43 logMAR after 3 months (*p* = 0.205).

After TE, it decreased significantly to 0.65 ± 0.69 logMAR at the 1st week (*p* = 0.001), was still decreased after 3 months to 0.38 ± 0.58 (*p* = 0.046) and regained baseline level at the 1st postoperative year (*p* = 0.194).

In both groups, no further significant changes of visual acuity occurred.

### Success rates

**Fig. 2:**
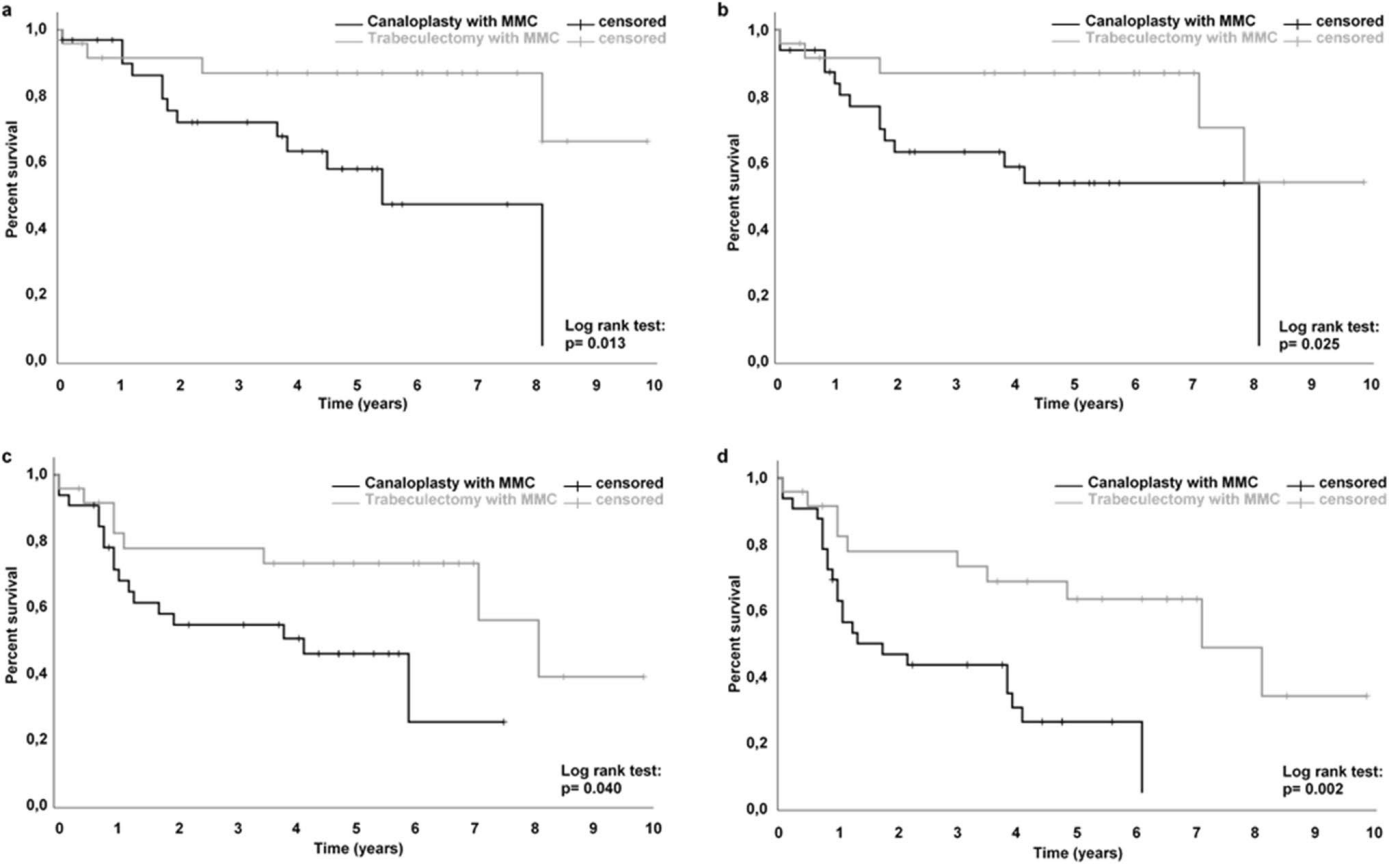
Success rates as Kaplan–Meier survival curves in criteria A, B, C and D after CP with MMC and TE with MMC. **a** no glaucoma revision surgery, **b** no glaucoma revision surgery, IOP < 21 mmHg, IOP reduction > 20%, **c** no glaucoma revision surgery, IOP < 18 mmHg, IOP reduction > 20%, **d** no glaucoma revision surgery, IOP ≤ 15 mmHg, IOP reduction ≥ 40%

Success rates are illustrated in Fig. 2. Eyes being lost due to non-appearance are shown as censored; eyes being lost due to exclusion by each criterion result in decrease of survival probability. After CP with MMC, success in criterion B was reached in 83% at the 1st year and 61% at 2nd and 3rd year. Thirteen glaucoma revision surgeries in 31 eyes were reported.

After TE with MMC, success in criterion B was reached in 91% at the 1st postoperative year and 86% at the 2nd and 3rd year. Four of 23 eyes received glaucoma revision surgeries.

Log rank test showed significant superiority of TE compared to CP until last follow-up in all four criteria.

According to the strongest criterion, D median time until failure after CP was 16 months (95%-CI: 6.5–25.5) and 85 months after TE (95%-CI: 46.3–123.7).

### Postsurgical complications

Postoperative complications are listed in Table 2. The most common complications were hyphema, astigmatism, hypertension, hypotony and bleb leakage.

**Table 2:** Early and late postsurgical complications

	Canaloplasty with MMC	Trabeculectomy with MMC	
Early (≤ 90 days) complications:	% (*n* = 31)	% (*n* = 23)	*p*-value
Hyphema	45%	30%	0.398
Bleb leakage	13%	30%	0.173
Haemorrhage beneath filtering zone	7%	26%	0.060
Corneal oedema	7%	13%	0.640
Corneal erosion	7%	0%	0.502
Self-parted synechia	3%	0%	1.000
Descemet membrane detachment	–	–	–
Intraocular inflammation	–	–	–
Early & late complications:	% (*n*)	% (*n*)	
Increased astigmatism > 1 dpt At 90 days	30% (20)	40% (15)	0.721
At 1st year	23% (22)	38% (16)	0.471
Hypotony ≤ 5 mmHg ≤ 90 days	16% (31)	44% (23)	0.035
≤ 1 year	4% (26)	16% (19)	0.295
≤ 2 years	5% (21)	8% (13)	1.000
Choroidal detachment/folds/swelling ≤ 90 days	7% (28)	17% (23)	0.390
Persistent > 90 days	0% (26)	10% (21)^a^	0.194
Hypertension ≥ 30 mmHg ≤ 90 days	19% (31)	48% (23)	0.039
≤ 1 year	8% (26)	0% (19)	0.501
≤ 2 years	14% (21)	0% (13)	0.270
Hypertensive or hypotonic events ≤ 7 days	16% (31)	65% (23)	0.003
> 7 until ≤ 90 days	28% (29)	43% (21)	0.366

^a^Persistent until 3rd and 4th year

### Ocular hypotony

After CP with MMC, ocular hypotony (≤ 5 mmHg) was measured in 5 eyes within the first 3 months (16%), equally distributed in medium and highly myopic eyes. All but one persistent hypotonic eye resolved to normotonic IOP until the 3rd month. Two eyes with low IOP showed choroidal detachment and choroidal swelling at the 7th day, but resolved until the 3rd month (7%, SE: − 6.0, − 7.5 dpt).

After TE with MMC 10 eyes had hypotony within the first 3 months (44%). Four of these needed surgical intervention like suturing of the scleral flap, compressive conjunctival sutures with collagen matrix or filtering bleb revision. Three eyes were hypotonic until the 1st year (16%). Choroidal folds and choroidal detachment were documented in 4 eyes with low to hypotonic IOP (17%). Two highly myopic eyes (− 7.125, − 18 dpt) persisted with macular folding until 3rd and 4th year, but none was long-term affected by decreased visual acuity.

Comparison between CP and TE shows significant higher occurrence of hypotony in eyes that received TE. No tendencies of higher occurrences in either the medium or highly myopic subgroups had statistical significance.

### Ocular hypertension

Postoperative hypertension was more frequent after CP than after TE. There were no significantly higher occurrences of hypertension in either the medium or highly myopic subgroups of CP or TE.

### IOP regulating early intervention

Early postsurgical treatment and interventions are presented in Table 3.

**Table 3:** Early and late postsurgical interventions

	Canaloplasty with MMC	Trabeculectomy with MMC	
	% (*n* = 31)	% (*n* = 23)	
Early (≤ 90 days) interventions:			*p*-value
Digital ocular massage	48%	70%	0.166
Suture lysis	61%	52%	0.583
Additional conjunctival sutures	3%	17%	0.151
Anterior chamber lavage	0%	4%	0.426
Scleral flap fixation	0%	4%	0.426
Eyes without any manipulation	36%	17%	0.026
Early and late interventions:			
Downsizing of bleb due to astigmatism/disturbance/pain	1 eye after 5 years	1 eye within 1st year	
Glaucoma revision surgery^f^			
≤ 90 days	3%^a^	4%^b^	
≤ 1st year	0%	5%^b^	
≤ 2nd year	29%^a,ccc,dd,e^	0%	
≤ 3rd year	0%	5%^b^	
≤ 4th year	13%^aa^	0%	

^a^TE with MMC

^b^Bleb revision with MMC

^c^Glaucoma drainage implants e.g., Ahmed Implant or Baerveldt Implant

^d^Combined cataract surgery with trabeculotomy

^e^Cyclophotocoagulation

^f^Defining failure of success rates in criterion A

The prevalence of intervention within the first 3 months was significantly higher after TE. Also, after TE, there was a tendency that the subgroup of highly myopic eyes needed more interventions as there were 13 of 14 highly myopic eyes (93%) compared to 6 of 9 medium myopic eyes (67%) (*p* = 0.293).

At the last follow-up, filtering zones were described as being present in 75% of CP patients and 91% of TE patients (*p* = 0.031).

## Discussion

Axial length is clinically considered a risk factor for surgical failure, although some studies of highly myopic eyes would not support this assumption [17]. The consideration of myopic eyes as a group that is vulnerable for complications subsequent to hyperfiltration [1, 4] is understood to be caused by the thinness of the sclera. It may provoke its collapse and therefore making choroidal folds and hypotony maculopathy more probable [18, 19]. CP with or without MMC is ranking between penetrating and minimally invasive glaucoma surgery considering both IOP reduction and risk profile. A study with a comparable cohort about solely highly myopic eyes receiving deep sclerectomy without MMC stated that IOP reduction was good and risk profile low [20]. Several reviews emphasize that the superiority of TE upon CP regarding success rates or efficacy is not significantly proven [5, 21].

In our single-surgeon study, we performed a retrospective analysis of operated myopic eyes, including subanalysis of medium and highly myopic subgroups.

Mean IOP did not differ relevantly in our group of myopic eyes compared to emmetrope cohorts in CP, also in long-term follow-up [10, 22, 23]. It was constantly lower after TE than after CP. Medication score reduction through CP is known to be weaker than through TE [5–7] and was seen in our analysis also clearly. Fittingly were the percentages of patients without any medication. These scores are relevant in their impact on patient’s comfort and in the interpretation of success rates, particularly as our analysis displays qualified success rates.

Hyphema was the most frequent complication after CP and is reported in lower to equal frequencies in most studies [9, 24]. It can cause postsurgical decrease of visual acuity or transient increase in IOP but is assumed not to have any negative effect on the long-term outcome. It is even regarded as a pointer for success as it is produced by reflux during manipulation of Schlemm’s canal [25]. The transient decrease of visual acuity after CP was mostly due to hyphema and astigmatism. After TE, the decrease lasted longer and might be caused by higher frequency and persistence of astigmatism.

One of the relevant complications in analysis was hypotony in the early and intermediate postoperative phase. The low incidence in our CP study group is in accordance to published data that report hypotony after CP without MMC in 9–20% [8, 9] or with MMC in 15% [26]. Congruently, the one highly myopic eye in our study with − 7.5 dpt that was persistently hypotonic until the 6th year had stable visual acuity until last follow-up and never showed signs of hypotonic pathologies in the posterior segment, just transient hypotonic calves of the cornea. The transient choroidal swelling and detachment in 2 eyes did not require intervention nor did it have any impact on visual acuity. Elsewhere, reported probabilities were comparable with 0–16% [7–9, 11, 26]. None of eyes with bleb leakage after CP was hypotonic at any time, and all resolved without intervention. As none of the hypotonic eyes had to receive surgical intervention, it could be termed as a clinically mild and self-resolving complication after CP with MMC in myopic eyes.

As expected through previous reports, TE with MMC showed significantly higher occurrence of hypotony in our study compared to CP and is equal to emmetrope study groups (20–42%) [8, 27, 28]. Seidel phenomenon was positive more frequently after TE (30%), and eyes were more likely to be hypotonic due to the leakage. Whereas, hypotony after CP was self-resorbing, after TE 4 eyes were treated surgically with intention to raise IOP.

We could not detect any relation between the degree of myopia and the occurrence of hypotony. Hypotony maculopathy was not seen in any eye in both patient groups. Still, the low incidence of hypotony-caused pathology of the posterior segment in our analysis is noteworthy; firstly, because of our myopic study group and secondly, because of our additional use of MMC.

Postsurgical hypertensive peaks were clearly contrary distributed in frequency and onset, as TE hypertensive peaks emerged early and CPs in later follow-up. It underlines the importance of close monitoring the first week after TE. Early hypertension after CP was successfully regulated with instant intervention through suture lysis and eyeball massage. Usually, these modulations can also be performed in an ambulant setting with IOP monitoring. As after TE only 4 eyes in total did not receive digital, laser or surgical intervention, treatment in the early postsurgical phase after CP was relevantly less frequent and invasive.

The presence of a filtering zone in CP patients may affect postoperative IOP level and success but our retrospective approach was not able to analyse this. As traditional CP is not intended to show a filtering zone, data is rare about the effect of postoperative interventions as suture lysis. The fact that in 75% of the eyes in our CP group some kind of a filtering zone was described in slit-lamp examination is not reflected in most studies about CP without additional use of MMC. In our cohort, one eye even received surgical downsizing of the filtering zone after 5 years.

In contrast to the early postoperative phase, hypertensions during later follow-up had to be solved through glaucoma revision surgery. Their rate after CP seemed to be relatively high in our cohort with 42% until last follow-up, which is reflected in the significant inferior success rates of criterion A. Success was significantly higher after TE than after CP in all graduated criteria. Success rates after CP without MMC in emmetrope cohorts are reported to be higher [7, 8, 10, 23] but also lower [29]. The high frequency of revision surgeries in our cohort could be related to the myopic study group. In their study about the development of postoperative hypertensive phases after implantation of glaucoma drainage devices, Jung and Park identified high myopia as risk factor. They are emphasizing the role of Tenon’s capsule in wound healing of the bleb in myopic eyes [30]. Nevertheless, the range of possible revision surgeries after CP does not seem to be severely impaired, as trabeculectomy or suture trabeculotomy with combined cataract surgery are among possible options [31]. Still, the decrease of success rates after CP has to be taken into account while deciding on the patient’s perspective with glaucoma as a lifelong disease.

### Limitations of this study

The retrospective analysis of CP and TE in myopic patients is vulnerable to bias in choice of surgical procedure, frequency of appearance in follow-up as well as heterogeneity of the patient’s ocular and glaucoma history. Until the 5th year, 21 of 31 patients after CP with MMC dropped out of analysis as did 10 of 23 eyes after TE with MMC. This is a relevant limitation to the statistical power of our findings. As these drop-outs are partly due to lack of follow-up data and are therefore treated as censored data in the survival curves, part of the loss is due to drop-out caused by failure according to criterion A. These eyes were excluded from further annual analyses. Nevertheless, survival curves could show significant and relevant results whereas the comparisons of mean IOPs in long follow-up have to be interpreted critically. The heterogeneity of the patients with regard on ocular and glaucoma history occurred because we did not set further exclusion criteria while choosing our myopic study groups than receiving the glaucoma surgery by the same surgeon in a consecutive series. Our aim was to find data as near as possible to a clinical routine setting of a commonly confronted to and relevant patient group. Nevertheless, the heterogeneity of our groups proved good concordance, and our findings were consistent to published data. Intraoperative application duration of MMC differed between the groups and could present a relevant influencing factor. Statements about complete success were not practicable, as indication on topical medication also lied in responsibilities of the resident doctors. In both groups, highly myopic patients made up ≥ 70% of the group. For this assembling and the limited group size itself, statistical power of subgroup analysis is low and especially conclusions about the relation between hypotony caused complications and the degree of myopia are critical. Future research would be suggested to undermine the worked-out tendencies of our study.

## Conclusion

In conclusion, we state that modified CP is a safe and effective surgical procedure to lower IOP in myopic and highly myopic glaucoma patients. Mean expected IOP levels are between 12 and 17 mmHg. IOP reduction after CP with MMC is slightly better in highly myopic patients. Medication score reduction < 2 in a mean is not lasting longer than 2 years. Incidence of hypotony is low; severe complications due to hypotony with impairment of the posterior eye’s segment is even lower. Recovery of visual acuity after CP is slightly faster. During years of follow-up revision, surgery is often required, but spectrum of subsequent surgery is not relevantly narrowed.

TE in myopic eyes proved to be a strong IOP lowering procedure to 10–13 mmHg with low incidence of hypo-tony in a long-term follow-up. Nevertheless, hypotonic and hypertonic crises were significantly higher than after CP. The need of surgical intervention in the early postsurgical phase was most likely. Revision surgery in long-term follow-up was required rarely.
